# Hemoperitoneum due to spontaneous bleeding from a smooth muscle tumor of uncertain malignant potential: A rare case report

**DOI:** 10.1016/j.ijscr.2023.107910

**Published:** 2023-01-31

**Authors:** Fotios Zachomitros, Ioannis Tsakiridis, Nikolaos Peitsidis, Georgios Michos, Themistoklis Dagklis, Ioannis Kalogiannidis

**Affiliations:** Third Department of Obstetrics and Gynaecology, Aristotle University of Thessaloniki, Greece

**Keywords:** Smooth muscle tumors of uncertain malignant potential, STUMP, Hemoperitoneum, Fibroid uterus

## Abstract

**Introduction and importance:**

Smooth muscle tumors of uncertain malignant potential (STUMPs) are uncommon tumors representing an extremely rare cause of hemoperitoneum.

**Case presentation:**

We report a case of a 48-year-old Caucasian, premenopausal woman that presented in the emergency department with acute abdominal pain. There was no remarkable past medical and surgical history except from a known uterine leiomyoma. The ultrasound and the computed tomography imaging showed an intraperitoneal fluid collection and a heterogenous uterine mass. The patient underwent emergent exploratory laparotomy; a subserosal uterine tumor was identified with an actively bleeding vessel on its surface. The uterine lesion was completely excised and the histopathology set the diagnosis of a STUMP. After consultation on the significance of this finding with the patient, an abdominal total hysterectomy and bilateral salpingo-oophorectomy were scheduled and performed and the subsequent histopathology detected no malignancy.

**Clinical discussion:**

This case demonstrates that a STUMP may be a rare cause of acute intraperitoneal bleeding. Careful evaluation of clinical history, imaging findings and, if needed, surgical exploration are important for the diagnosis, while appropriate follow-up is also of major importance for the management of these rare tumors.

**Conclusion:**

We presented an extremely rare case of hemoperitoneum due to spontaneous bleeding from a STUMP. From an oncological perspective, this case poses a diagnostic, management and follow-up challenge.

## Highlights

• STUMPs represent a rare type of uterine tumors.
• STUMPs may be an extremely rare cause of intraperitoneal bleeding.
• There is no consensus on the appropriate management of STUMPs, but close follow-up should be considered.

## 1 Introduction

Leiomyomas are benign tumors exhibiting various forms of smooth muscle differentiation and represent the most common uterine tumors [1]. Many studies have used the terms leiomyoma with bizarre nuclei and smooth muscle tumors of uncertain malignant potential (STUMPs) interchangeably under the term “atypical leiomyoma” [2]. With regards to STUMPs, they are tumors of unknown prevalence that cannot be classified as benign or malignant [3]. Spontaneous rupture of leiomyomas or STUMPs is an extremely rare cause of hemoperitoneum [4]. We report a unique case of rupture of a STUMP with the aim to contribute to the limited experience of clinical behavior of this type of uterine tumors. This case report has been written in accordance to the SCARE criteria [5].

## 2 Presentation of the case

A 48-year-old Caucasian woman was diagnosed with a uterine tumor of 4 cm maximum diameter during the investigation for minor constipation complains. The woman was otherwise asymptomatic, thus routine follow-up with transvaginal ultrasound (TVUS) by a gynecologist was recommended. Regarding her medical history, she reported two vaginal deliveries in the past, did not take any medications and her menstrual period was regular without history of abnormal uterine bleeding or dysmenorrhea. She reported herself as being a current social smoker and alcohol drinker.

Six months later, she presented at the emergency department due to acute abdominal pain. On physical examination, she had mild pallor and no significant lymphadenopathy. Her vital signs were normal apart from current tachycardia (pulse: 110/min), indicative of possible hypovolemic status. Abdominal examination revealed distension of lower abdomen with tenderness. On speculum examination, cervix and vagina were normal, while the cervical manipulation was tender. The pregnancy test was negative and TVUS along with transabdominal ultrasound showed mildly hyperechoic fluid in the peritoneal cavity and especially in the pouch of Douglas. There was also a significant drop in the hemoglobin (from 12.0 g/dL to 9.0 g/dL).

**Fig. 1:**
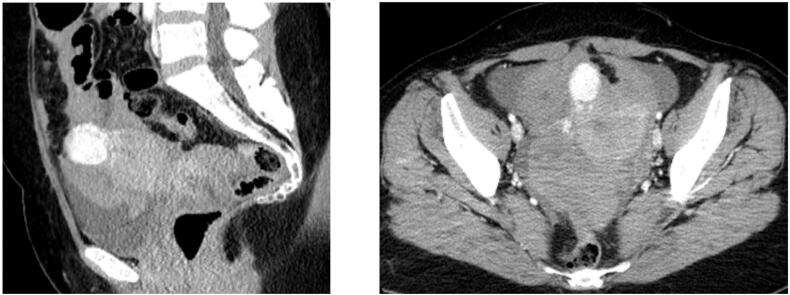
CT imaging showing the uterine tumor with an active bleeding area.

She was admitted for close monitoring. Her pulse and blood pressure remained stable, while abdominal distension and pallor gradually increased. She received two liters of colloids and crystalloids and two units of blood. Moreover, she underwent a computed tomography (CT) imaging, which revealed an intraperitoneal fluid collection and a heterogenous uterine mass (Fig. 1). Within 6 h, despite the transfusion, the hemoglobin decreased to 8.8 g/dL, so a decision for emergency exploratory laparotomy was taken. A subserosal uterine tumor FIGO type 6 [6], was identified and characterized macroscopically as a leiomyoma. An actively bleeding vessel on its surface was recognized as the origin of bleeding. The uterine mass was excised, and almost 2 L of blood and clots were evacuated from the peritoneal cavity. The patient's postoperative course was unremarkable.

**Fig. 2:**
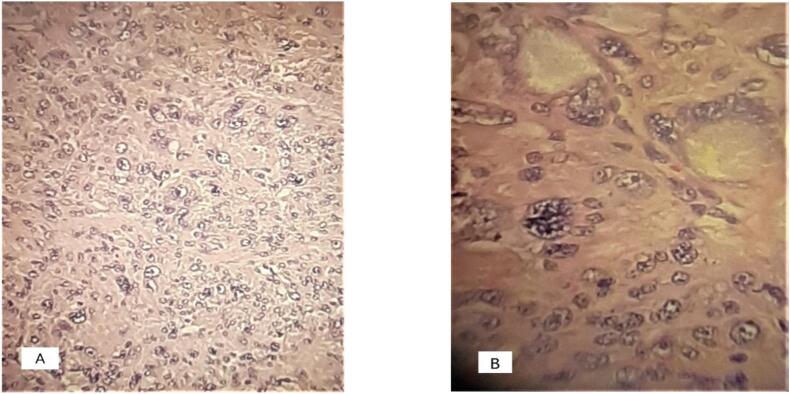
A) Atypical cells between fascicles of smooth muscle cells, hematoxylin and eosin stain 20× B) atypical cells 40×.

The histopathology set the diagnosis of a STUMP (Fig. 2); the tumor had diffuse moderate to severe atypia, no tumor cell necrosis and mitotic index <4 per 10/high-power field (HPF). The patient was counselled regarding the possibility of future recurrence of STUMP or leiomyosarcoma. She opted for further management and was readmitted three months later for the final surgical treatment; an abdominal total hysterectomy with bilateral salpingo-oophorectomy was performed, as the woman did not desire fertility preservation. The final histology showed no malignancy of the uterine body, cervix, fallopian tubes and ovaries.

## 3 Discussion

The clinical signs of STUMPs may include pain or pelvic pressure and abnormal uterine bleeding that can cause anemia [7]. Our case is unique due to the following aspects: first, the tumor was considered to be leiomyoma by the CT imaging, second, the tumor caused hemoperitoneum and an emergency laparotomy was needed. In addition, the tumor was only 4 cm in diameter, which is in contrast to the theory that only tumors >10 cm in diameter may be associated with stretching and tension of the overlying vessels resulting in rupture [8].

Our case shows that a 48-year-old premenopausal woman without family history of malignancy and an apparently small leiomyoma-like tumor do not exclude a diagnosis of STUMP. In fact, a previous report showed that the mean age of these patients was 43 years [9]. Moreover, STUMPs are extremely rare in postmenopausal women [10].

A preoperative diagnosis of hemoperitoneum associated with leiomyomas is not common without prior knowledge of the tumor; imaging techniques, such as CT or magnetic resonance imaging (MRI), may not be able to distinguish with accuracy the presence of malignancy or the subtypes of benign uterine tumors [11]. Notably, pelvic ultrasonography is the suggested imaging modality for the early diagnosis of uterine tumors [12].

Acute abdominal pain associated with hypovolemia and hemodynamic instability often necessitates emergency laparotomy [13]. In our case, the history of a previously diagnosed uterine tumor was the main determinant of early admission, urgently conducted diagnostic examinations and laparotomy. The recommended treatment approach for these lesions includes tumor excision or hysterectomy in women not desiring fertility preservation [14]. The rate of recurrence of STUMPs can be up to 28 % and, in rare cases, the tumor may progress to a leiomyosarcoma [3], [15].

Histologically, the diagnosis of a leiomyosarcoma requires at least two of the Stanford criteria: diffuse moderate-to severe atypia, mitotic count ≥10 mitotic figures/10 HPF and tumor cell necrosis. The diagnosis of a STUMP, on the other hand, is also based on the above-mentioned criteria, but without fulfilling the diagnosis of a leiomyosarcoma [3].

Our follow-up plan was to counsel the patient regarding the prognosis and discuss the management options. As already mentioned, the patient did not desire fertility preservation. The optimal follow-up of such patients, especially after a conservative approach, has not been determined, however, a time interval of six months to five years has been suggested [16]. In addition, the optimal method (clinical, imaging, laboratory) of follow-up is also yet to be determined [17].

## 4 Conclusion

We presented an extremely rare case of hemoperitoneum due to spontaneous bleeding from a STUMP. Such a manifestation with bleeding is extremely rare and once acute abdomen is encountered, preoperative diagnosis of internal bleeding due to the presumed leiomyoma might be obscured. Our case demonstrates that medical history, imaging and surgical exploration are useful to identify the source of bleeding and prevent a potentially fatal outcome. This case poses a diagnostic, management and follow-up challenge from an oncological perspective.

## Patient consent

Written informed consent was obtained from the patient for publication of this case report and accompanying images. A copy of the written consent is available for review by the Editor-in-Chief of this journal on request.

## Ethical approval

This article does not contain any personal information that can lead to the identification of the patient. Ethical approval is exempt/waived at our institution for this type of articles.

## Funding sources

This research did not receive any specific grant from funding agencies in the public, commercial, or not-for-profit sectors.

## Author contribution

Zachomitros Fotios: Paper design, paper writing.

Tsakiridis Ioannis: Paper design, data collection, paper writing.

Peitsidis Nikolaos: Paper design, data collection, paper writing.

Michos Georgios: Paper review, Picture preparation.

Dagklis Themistoklis: Picture preparation, paper review.

Kalogiannidis Ioannis: Paper design, data collection, paper review.

## Guarantor

Ioannis Tsakiridis, Ioannis Kalogiannidis.

## Research registration number

N/A.

## Declaration of competing interest

The authors have no conflict of interest to declare.
